# Sensing Properties of a Novel Temperature Sensor Based on Field Assisted Thermal Emission

**DOI:** 10.3390/s17030473

**Published:** 2017-02-27

**Authors:** Zhigang Pan, Yong Zhang, Zhenzhen Cheng, Jiaming Tong, Qiyu Chen, Jianpeng Zhang, Jiaxiang Zhang, Xin Li, Yunjia Li

**Affiliations:** 1 State Key Laboratory of Electrical Insulation and Power Equipment, Xi’an Jiaotong University, Xi’an 710-049, China; panzhigang0703@126.com (Z.P.); celia111@stu.xjtu.edu.cn (Z.C.); tongjiaming@stu.xjtu.edu.cn (J.T.); cqy837879002@sina.com (Q.C.); zhangjp22@stu.xjtu.edu.cn (J.P.Z.); zjx972200751@stu.xjtu.edu.cn (J.X.Z.); liyunjia@xjtu.edu.cn (Y.L.); 2 Vacuum Micro-Electronic & Micro-Electronic Mechanical Institute, School of Electronics and Information Engineering, Xi’an Jiaotong University, Xi’an 710049, China; lx@mail.xjtu.edu.cn

**Keywords:** temperature sensor, carbon nanotubes, ionization, emission

## Abstract

The existing temperature sensors using carbon nanotubes (CNTs) are limited by low sensitivity, complicated processes, or dependence on microscopy to observe the experimental results. Here we report the fabrication and successful testing of an ionization temperature sensor featuring non-self-sustaining discharge. The sharp tips of nanotubes generate high electric fields at relatively low voltages, lowering the work function of electrons emitted by CNTs, and thereby enabling the safe operation of such sensors. Due to the temperature effect on the electron emission of CNTs, the collecting current exhibited an exponential increase with temperature rising from 20 °C to 100 °C. Additionally, a higher temperature coefficient of 0.04 K^−1^ was obtained at 24 V voltage applied on the extracting electrode, higher than the values of other reported CNT-based temperature sensors. The triple-electrode ionization temperature sensor is easy to fabricate and converts the temperature change directly into an electrical signal. It shows a high temperature coefficient and good application potential.

## 1. Introduction

Temperature sensors are among the most-widely used sensors in consumer and industrial temperature measurement. The development goal of a temperature sensor usually includes high sensitivity, small footprint, and low power consumption. The potential of a carbon nanotubes (CNTs) based temperature sensor offers great opportunities towards extreme miniaturization and low power consumption [1,2,3,4,5], thanks to the unique nanoscale structure and electrical property [6,7,8,9,10,11]. Recent studies showed that CNTs could be used to construct temperature sensors. For example, a thermometer can be realized by measuring the thermal expansion of gallium inside a CNT [1], since the height of a continuous unidimensional column of liquid gallium inside a carbon nanotube varies linearly and reproducibly in the temperature range 50–500 °C. Nevertheless, this methodology requires microscopic measurement of the height of the gallium. Alternatively, the CNT-based temperature sensors [2,3,4,5] can also be implemented by measuring the conductivity variation of CNT induced by the thermal interaction. However, the application of this type of sensor is potentially limited by its complex fabrication process and low sensitivity. A novel ionization temperature sensor based on CNTs electrodes [12] was capable of overcoming the limitations of the above two types of temperature sensors, but it was only used to detect temperature in N_2_ at 100 V extracting voltage; meanwhile, its carbon nanotubes have large diameter and small spaces between nanotubes (see supplementary material Figure S1), which results in small field enhancement factor, small output current, and low sensitivity—especially below 70 °C (see supplementary material Tables S1 and S2, Figure S2). In this paper, a triple-electrode CNT-based ionization temperature sensor is fabricated. Differently, the CNTs array is grown by a low-temperature thermal chemical vapor deposition (TCVD) process, enabling small diameters and larger spaces between nanotubes. As a result, the electric field around CNT tips and the emission current density of the tips can be enhanced [13]. In addition, the decrease of the electrode separation can increase the electric field strength of the sensor. The fabricated CNT temperature sensor is capable of detecting temperature in ambient atmosphere at low working voltage. The temperature sensing mechanism of the CNT sensor is explained in terms of the emission of CNT and the discharge properties of air.

## 2. Materials and Methods 

**Figure 1:**
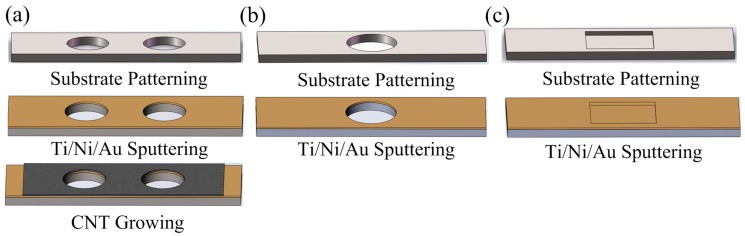

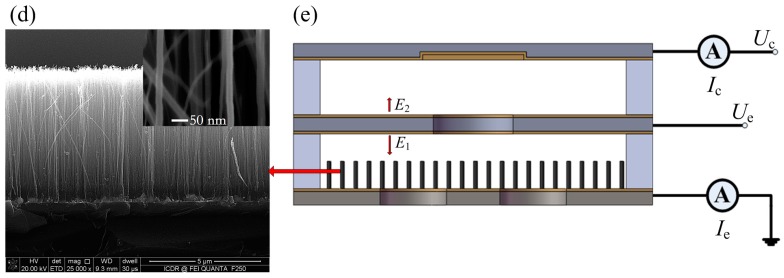
Scheme of the triple-electrode carbon nanotube (CNT)-based sensor. (**a**) the cathode after substrate patterning, Ti/Ni/Au sputtering, and CNT growing; (**b**) the extracting electrode and (**c**) the collecting electrode after substrate patterning and Ti/Ni/Au sputtering; (**d**) Scanning electron microscope image of the CNT film; (**e**) The test set-up.

Figure 1 shows the schematic illustration of the presented CNT-based temperature sensor. The sensor is comprised of three electrode plates; i.e., a CNT-based cathode, an extracting electrode, and a collecting electrode. The two ventilating holes in the cathode make the gas diffuse more easily [11]. A hole in the extracting electrode is used to extract discharge particles. A rectangular ditch in the collecting electrode could reduce reflection-induced loss of positive ions and collect more. In operation, the extracting voltage *U*_e_ is set higher than the collecting voltage *U*_c_, where the potential of the cathode is 0 V. In this configuration, two electric fields *E*_1_ and *E*_2_ are generated with reversed field direction. The currents *I*_c_ and *I*_e_ were recorded by two high-precision digital multimeters (NI PXI-4071, National Instruments Corporation, Austin, TX, USA). One minute after the voltages are applied to the electrodes and the discharge of air is stable, ten current values of *I*_c_ and ten current values of *I*_e_ are recorded and averaged as currents *I*_c_ and *I*_e_, respectively.

The presented CNT temperature sensor was fabricated according to the following steps. Three Si substrates were firstly patterned by photolithograph to form the pattern of the cavity with different sizes. The patterned substrates were then etched by an inductively coupled plasma (ICP) etcher. The etched structure included the cathode with two circular holes of 4 mm in diameter, the extracting electrode with a circular hole of 6 mm in diameter, and the collecting electrode with a rectangular ditch structure with the size of 8 mm × 6 mm × 200 µm (length × width × depth) (Figure 1a–c). A metallization layer of Ti/Ni/Au was then sputtered on both sides of the extracting electrode and the inner walls of the cathode and collecting electrode. Vertically aligned multi-walled carbon nanotubes (MWCNTs) were subsequently grown by thermal chemical vapor deposition (TCVD) on one side of the cathode at 700 °C [14], with around 20 nm in diameter, 5 μm in length, and separated with a distance of 200 nm in between (Figure 1d). The three electrodes were assembled with 50 µm-thick polyester films. 

## 3. Results

**Figure 2:**
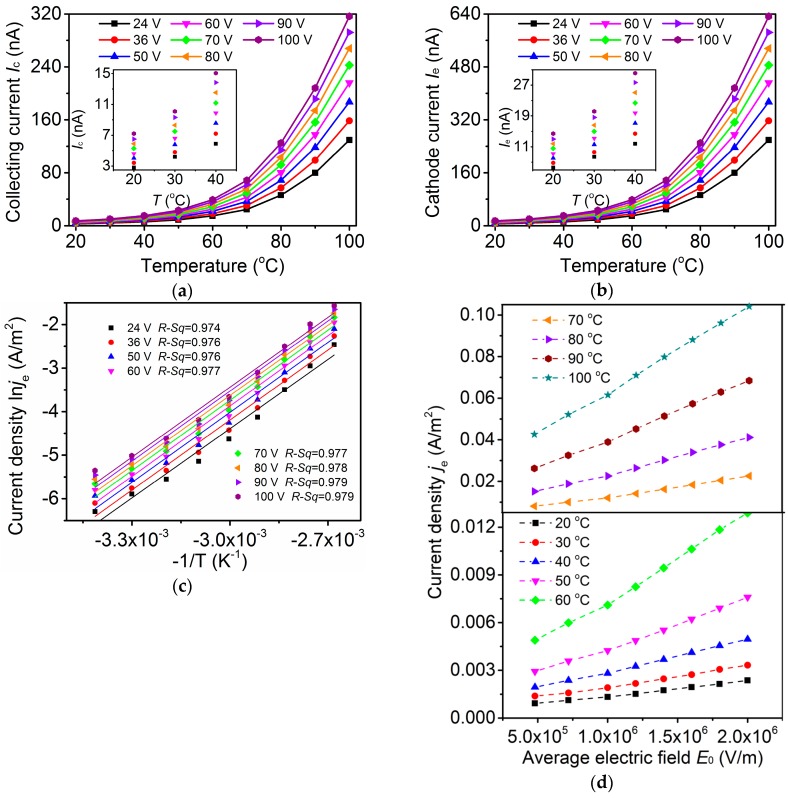
Effect of temperature on current at different *U*_e_. (**a**) Collecting current–temperature characteristic and (**b**) Cathode current–temperature characteristic at 24–100 V *U*_e_; (**c**) ln*j*_e_ increased linearly with −1/*T* at 24–100 V *U*_e_, suggesting the thermal emission behavior and a considerable effect of temperature on current density; (**d**) *j*_e_ increased with *E*_0_, suggesting the field emission behavior.

The effect of temperature on the discharge current of air was studied as depicted in Figure 1e. The fabricated sensor was placed inside a test chamber filled with ambient air heated by a resistive wire. The temperature inside the chamber was closed-loop controlled by tuning the voltage of the resistive heating wire and measuring the temperature using a k-type thermocouple (chromel–nisiloy). The steady state of the temperature was realized within the time of 1 min, in a range of 20 °C to 100 °C. After the temperature reached steady state, *U*_c_ of 1 V and *U*_e_ with values between 24 and 100 V were applied, and the discharge current *I*_c_ and *I*_e_ were measured respectively for the detection temperature and the study of emission properties of the CNTs cathode. When temperature *T* rose from 20 °C to 100 °C, currents *I*_c_ and *I*_e_ exhibited an exponential increase with *T* (Figure 2a,b). When extracting voltage *U*_e_ increased from 24 V to 100 V, currents *I*_c_ and *I*_e_ exhibited an increase with *U*_e_. Sensitivity curves to temperature show very similar shape, as shown in Figure 2. This indicates that the sensor is sensitive to temperature and is thus capable of detecting temperature change. Values of *I*_e_ were almost twice of those of *I*_c_. The temperature coefficients of Figure 2a were calculated according to equation *S* = Δ*I*/(Δ*T*·*I*_FS_), where Δ*T* is the variation of temperature, Δ*I* is the variation of *I*_c_, and *I*_FS_ is the full scale range of *I*_c_. The highest coefficient *S*_max_ was obtained as 0.04 K^−1^ at 100 °C and 24 V *U*_e_ (Table 1), higher than the values of other reported CNT-based temperature sensors [1,2,3,4,5].

The relationship between the logarithm ln*j*_e_ (*j*_e_ is the cathode current density and is obtained by *j*_e_ = *I*_e_/*S*_area_) and the reciprocal –1/*T* was studied (Figure 2c). *S*_area_ is the total cross-sectional area of all nanotubes on the film, and was calculated according to Figure 1d (Figure S3 in reference [15]); *S*_area_ = 3.04 × 10^−6^ m^2^. Eight least squares straight lines were fitted to the curves of ln*j*_e_−1/*T* at different *U*_e_, and the coefficients *R*-*Sq* were calculated (Figure 2c)

*R-Sq* = 1 − (Σ(*y*_i_ − *f*_i_)^2^)/(Σ(*y*_i_ − *y*_av_)^2^) (1)

where *y*_i_ denotes ln*j*_e_, *f*_i_ denotes the fitting value to ln*j*_e_, *y*_av_ denotes the mean value of all ln*j*_e_, and subscript *i* denotes the sequence number. The coefficients *R*-*Sq* show that the straight lines are the best fit line to the curves at various *U*_e_, indicating a thermal emission behavior of the sensor. The relationship between current density *j*_e_ and the average electric field *E*_0_ was also obtained (Figure 2d), *E*_0_ = *U*_e_/*d*, where *d* denotes electrode separation between cathode and extracting electrode. *j*_e_ increased with *E*_0_, showing a field-assisted thermal emission behavior [16].

## 4. Discussion

**Figure 3:**
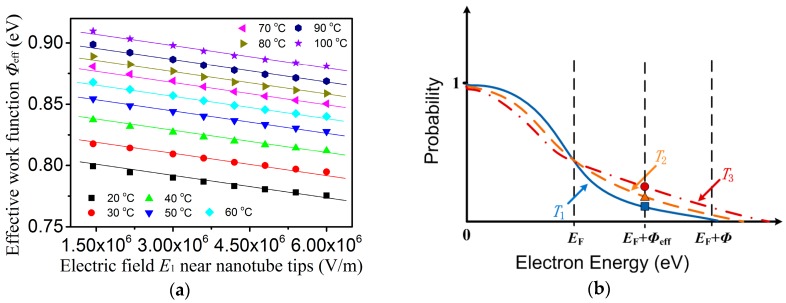
(**a**) The effects of the *E*_1_ on *Φ*_eff_; (**b**) The effect of temperature on the probability of an electron gaining energy.

This section analyzes the experimental results to understand the temperature sensing mechanism of the present CNT temperature sensor. When *U*_e_ is applied, electrons are emitted from the nanotube tips and collide with the gas molecules, generating positive ions [17]. A fraction of the generated positive ions are extracted from the cathode region through the extracting hole towards the collecting electrode and form the collecting current *I*_c_. Consequently, *I*_c_ is a part of the discharge current *I* [18], given by:

*I = eN*_0_*e^α^^d^* (2)

where *e* is the electron charge, *N*_0_ is the number of electrons leaving the cathode in one second, and *α* is the first ionization coefficient. Emission electrons and secondary emission electrons of the CNT cathode contribute to *N*_0_. In the non-self-discharge state, *N*_0_ is mainly determined by the emission electrons [18]. It is known that only those electrons with energies greater than the sum of Fermi energy *E*_F_ and work function *Φ* can be emitted by the carbon nanotubes. When *U*_e_ is applied, the work function of electrons emitted by the CNT is reduced from *Φ* to *Φ*_eff_. *Φ*_eff_ is the effective work function, and it decreased with increasing *E*_1_ at a given temperature (Figure 3a). More electrons with energies greater than *E*_F_ + *Φ*_eff_ could be emitted by CNTs. It is well known that when temperature rises to *T*_2_ and *T*_3_ from *T*_1_ (100 °C ≥ T_3_ > *T*_2_ > *T*_1_), respectively, the probability of an electron gaining more energy increases (Figure 3b) [16]. As a result, more electrons leave the cathode at higher temperature and higher *U*_e_, and form larger emission current*I*_0_ (*I*_0_= *eN*_0_) [19].The emission current density *J*_Schottky_ could be expressed as follows [16]:

*J*_Schottky_*= B*_0_*T*^2^*exp(−Φ*_eff_*/kT)* (3)

where *B*_0_ is the Richardson constant and *k* is the Boltzmann constant. Since *J*_Schottky_ is the main source of *j*_e_, it is approximated in this work that *J_Schottky_* ≈ *j*_e_, and exponential increase *I*_e_ with temperature could be obtained. The higher the temperature and *U*_e_ are, the larger are the cathode currents. The experimental results in Figure 2 are in good agreement with the above analysis.

Additionally, the first ionization coefficient *α* was also affected by temperature [17],

*α = exp(–U_i/_(Eλ))/λ* (4)

where *λ* is the mean free path of an electron, *U*_i_ is the first ionization potential of a gas molecule, and *E* is electric field.

*λ = kT/(πr^2^P)* (5)

where *k* is the Boltzmann constant, *r* is the radius of gas molecules, *P* is gas pressure. *α* denotes the impact ionization ability of an electron with a gas molecule, which depends on *E*, *λ,* and *U*_i_. Here, *E* does not change when electrode separation and applied voltage are given, and the first ionization potential *U*_i_ is a constant for a certain gas. As a result, *α* changes with *λ*. Since pressure *P* increases with temperature *T* at a fixed volume of the test chamber, *λ* does not change with temperature according to Equation (5). Therefore, rising temperature could not affect *α* in the experiment of this work. If the temperature sensor operates in an open environment, *λ* increases with temperature *T* at constant pressure *P*, and then the effect of temperature on *α* and discharge current should be considered.

## 5. Conclusions

In summary, a triple-electrode CNT-based ionization sensor was fabricatedby using micro fabrication technology. A vertically aligned multi-walled nanotube array was grown by thermal chemical vapor deposition on one side of the cathode. The relationship of currents *I*_c_ and *I*_e_ versus temperature was investigated in a wider test range of 20–100 °C and at 24–100 V *U*_e_. The sensor in this work had a sensitivity of 0.04 K^−1^ at 24 V *U*_e_, which is the highest sensitivity compared to the existing CNT-based resistive temperature sensors. The discharge current *I*_e_ and *I*_c_ increased with temperature, due to the increased number of electron emission at a given volume of gas mixture.

## Figures and Tables

**Table 1:** Performance comparison of the temperature sensors.

No.	Test Range (°C)	Highest Temperature Coefficient (K^−1^)	Reference
This paper	20–100	4.0 × 10^–2^	-
Article 1	50–500	2.2 × 10^–3^	[1]
Article 2	−269–147	−7.0 × 10^–4^	[2]
Article 3	20–70	−1.3 × 10^–2^	[3]
Article 4	20–75	−1.7 × 10^–2^	[4]
Article 5	20–60	−5.0 × 10^–3^	[5]

## Supplementary Materials

The Supplementary Materials are available online at http://www.mdpi.com/1424-8220/17/3/473/s1.

## References

[1] Gao Y.H., Bando Y. (2002). Carbon nanothermometer containing gallium-Gallium’s macroscopic properties are retained on a miniature scale in this nanodevice. Nature.

[2] Bartolomeo A.D., Sarno M., Giubileo F., Altavilla C., Iemmo L., Piano S., Bobba F., Longobardi M., Scarfato A., Sannino D., Cucolo A.M., Ciambelli P. (2009). Multiwalled carbon nanotube films as small-sized temperature sensors. J. Appl. Phys..

[3] Karimov K.S., Chani M.T.S., Khalid F.A. (2011). Carbon nanotubes film based temperature sensors. Phys. E.

[4] Karimov K.S., Khalid F., Chani M., Mateen A., Hussain M.A., Maqbool A., Ahn J. (2012). Carbon nanotubes based flexible temperature sensors. Optoelectron. Adv. Mater.-Rapid Commun..

[5] Matzeu G., Pucci A., Savi S., Romanelli M., Di Francesco F. (2012). A temperature sensor based on a MWCNT/SEBS nanocomposite. Sens. Actuators A.

[6] Walters D.A., Ericson L.M., Casavant M.J., Liu J., Colbert D.T., Smith K.A., Smalley R.E. (1999). Elastic strain of freely suspended single-wall carbon nanotube ropes. Appl. Phys. Lett..

[7] Li W.Z., Wen J.G., Ren Z.F. (2001). Straight carbon nanotube Y junctions. Appl. Phys. Lett..

[8] Cadek M., Coleman J.N., Barron V., Hedicke K., Blau W.J. (2002). Erratum: “Morphological and mechanical properties of carbon-nanotube-reinforced semicrystalline and amorphous polymer composites”. Appl. Phys. Lett..

[9] Modi A., Koratkar N., Lass E., Wei B.Q., Ajayan P. M. (2003). Miniaturized gas ionization sensors using carbon nanotubes. Nature.

[10] Zhang Y., Liu J., Li X., Tang X.J., Zhu C.C. (2005). Study of improving identification accuracy of carbon nanotube film cathode gas sensor. Sens. Actuators A.

[11] Zhang Y., Liu J., Li X., Zhu C.C. (2006). The structure optimization of the carbon nanotube film cathode in the application of gas sensor. Sens. Actuators A.

[12] Zhang Y., Li S.T., Zhang J.Y., Pan Z.G., Min D.M., Li X., Song X.P., Liu J.H. (2013). High-performance gas sensors with temperature measurement. SCI REP-UK.

[13] Passacantando M., Bussolotti F., Santucci S., Bartolomeo A.D., Giubileo F., Iemmo L., Cucolo A.M. (2008). Field emission from a selected multiwall carbon nanotube. Nanotechnology.

[14] Li X., Liu J.H., Dou J.Y., Liu W.H., Zhu C.C. (2002). Improvement of purity and field emission character of carbon nanotubes film by optimizing the density of thecatalyst solution. Chin. J. Xi’an Jiaotong Univ..

[15] Zhang J., Zhang Y., Pan Z.G., Yang S., Shi J.H., Li S.T., Min D.M., Li X., Wang X.H., Liu D.X., Yang A.J. (2015). Properties of a weakly ionized NO gas sensor based on multi-walled carbon nanotubes. Appl. Phys. Lett..

[16] Kasap S.O. (2002). Principles of Electronic Materials and Devices.

[17] Bartolomeo A.D., Scarfato A., Giubileo F., Bobba F., Biasiucci M., Cucolo A.M., Santucci S., Passacantando M. (2007). A local field emission study of partially aligned carbon-nanotubes by atomic force microscope probe. Carbon.

[18] Raizer Y.P., Allen J.E. (1991). Gas Discharge Physics.

[19] Ahmed S.F., Das S., Mitra M.K., Chattopadhyay K.K. (2007). Effect of temperature on the electron field emission from aligned carbon nanofibers and multiwalled carbon nanotubes. Appl. Surf. Sci..

