# Supplementary material for: Sensing Properties of a Novel Temperature Sensor Based on Field Assisted Thermal Emission
Source: Sensors (Basel). 2017 Feb 27;17(3):473. doi: 10.3390/s17030473 (PMC5375759; doi:10.3390/s17030473)
Supplement: Supplementary file 1 [file sensors-17-00473-s001.pdf]

Supplementary Material

Sensing Properties of a Novel Temperature Sensor Based on Field Assisted Thermal Emission

Zhigang Pan [1], Yong Zhang [1,*], Zhenzhen Cheng [1], Jiaming Tong [1], Qiyu Chen [1], Jianpeng Zhang [1], Jiaxiang Zhang [1], Xin Li [2], and Yunjia Li [1]

[1] State Key Laboratory of Electrical Insulation and Power Equipment, Xi'an Jiaotong University, Xi'an, 710-049, China; panzhigang0703@126.com (Z.P.); celia111@stu.xjtu.edu.cn (Z.C.); tongjiaming@stu.xjtu.edu.cn (J.T.); cqy837879002@sina.com (Q.C.); zhangjp22@stu.xjtu.edu.cn (J.Z.); zjx972200751@stu.xjtu.edu.cn (J.Z.); liyunjia@xjtu.edu.cn (Y.L.)

[2] Vacuum Micro-Electronic & Micro-Electronic Mechanical Institute, School of Electronics and Information Engineering, Xi'an Jiaotong University, Xi'an, 710049, China; lx@mail.xjtu.edu.cn

* Correspondence: zhyong@mail.xjtu.edu.cn; Tel.: +86-29-8266-8793

1. Comparison between the shape and distribution of MWCNTs fabricated in the paper and reference [12]

The multi-walled carbon nanotubes (MWCNTs) array was grown by thermal chemical vapor deposition (TCVD) method. In reference [12], the CNTs were grown at 850 ^{o}C, with ~50 nm in diameter, ~5 μm in length, and ~100 nm in separation between nanotubes (Figure S1a). In the paper, the CNTs were grown at 700 ^{o}C, with ~20 nm in diameter, ~5 μm in length, and ~200 nm in separation between nanotubes (Figure S1b). Because the thinner nanotip and larger separation between nanotubes could lead to a higher field enhancement factor (Table S1), the MWCNTs in the paper could enhance the strength of the electric field near the nanotips and increase the current density.

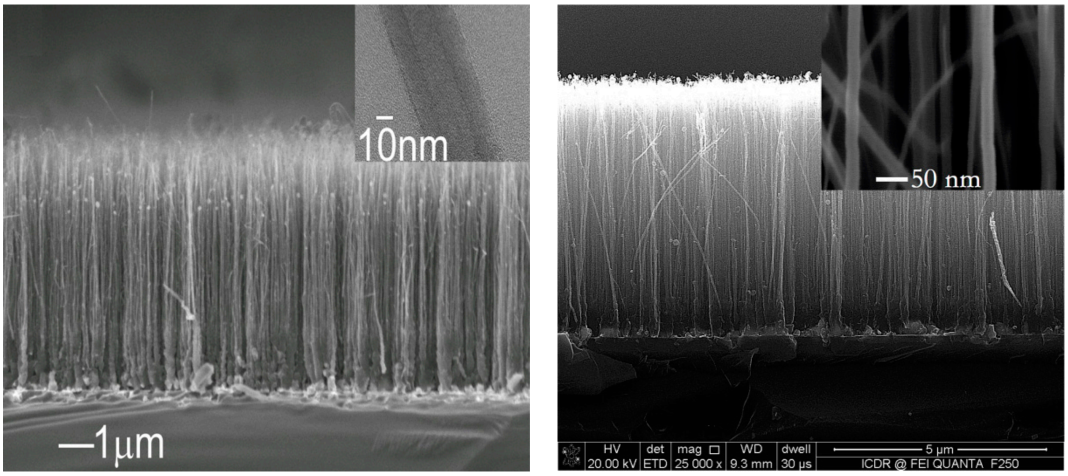

(a) (b)

Figure S1. SEM images of a thermal chemical vapor deposition (TCVD)-grown, vertically aligned multi-walled carbon nanotube (MWCNT) film in (**a**) reference [12] and (**b**) this paper, respectively.

Table S1. Field enhancement factor comparison of the MWCNT film.

No.	Field enhancement factor λ^{a} near carbon nanotube tip
This paper	32.94
Ref. [12]	4.26

[a] The field enhancement factor $\lambda = 3+2(1+\rho/h)/\{(2+\rho/h)[2\pi(2+\rho/h)(\rho/D)^2+\rho/h]\}$ [1], ρ is the radius of a carbon nanotube, h is the height of a carbon nanotube, and D is the distance between the nearest carbon nanotube.

[1] A. I. Zhbanov, E. G. Pogorelov, Y. C. Chang, Y. G. Lee, Screened field enhancement factor for the floating sphere model of a carbon nanotube array, J Appl Phys 110 (2011) 114311.

2. Experimental collecting current-temperature characteristic of the temperature sensors in the paper and the reference [12]

We chose temperature sensor with 50 μm separations in the paper and another temperature sensor with 170 μm separations in reference [12], and conducted tests for detecting temperature in a 20-100 ℃ range at 70 V U_e shown in Figure S2. The temperature sensor in the paper shows a higher sensitivity than that of the reference [12] shown in Table S2. The temperature coefficients of Figure S2 is calculated according to the equation $S = \Delta I / (\Delta T \cdot I_{FS})$, where ΔT is the variation of temperature, ΔI is the variation of I_c and I_{FS} is the full scale range of I_c.

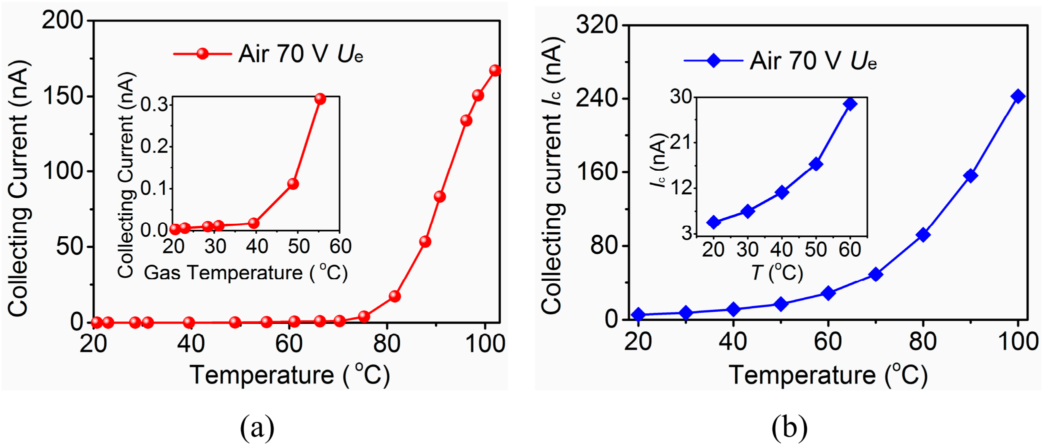

Figure S2. Collecting current-temperature characteristic of the sensors in the reference [12] (a) and this paper (b), respectively.

Table S2. Performance comparison of the temperature sensors.

No.	Operating voltage (V)	Test range (℃)	Highest temperature coefficient (K^{-1})
This paper	70	20–100	1.12×10^{-3}
Ref. [12]	70	20–100	6.50×10^{-4}
